# The Effect of a Mindfulness-Based Education Program on Brain Waves and the Autonomic Nervous System in University Students

**DOI:** 10.3390/healthcare9111606

**Published:** 2021-11-22

**Authors:** Mijung Jung, Mikyoung Lee

**Affiliations:** Department of Nursing, Kwangju Women’s University, Gwangju 62396, Korea; mijeong@kwu.ac.kr

**Keywords:** mindfulness, mindfulness-based education program, brain waves, autonomic nervous system, university students

## Abstract

Background: Mindfulness, defined as the awareness emerging from purposefully paying attention to the present moment, has been shown to be effective in reducing stress and, thus, promoting psychological well-being. This study investigated the effects of a mindfulness-based education program on mindfulness, brain waves, and the autonomic nervous system (ANS) in university students in Korea. Methods: This study is a quantitative and experimental research with a single-group pre-post design. Six sessions of mindfulness-based intervention were applied. In total, 42 students completed a mindfulness questionnaire before and after the intervention, and 28 among them completed pre-intervention and post-intervention measures of brain waves and ANS. Results: The level of mindfulness increased in the participants after intervention. Regarding brain waves, the alpha and theta waves increased, but the beta waves decreased. There was no significant difference in the ANS, presenting no change in heart rate variability. Conclusions: We identified the positive effects of the mindfulness-based education program for university students. The findings indicate that this program may help students not only relax, but also generate a mindfulness state in stressful situations, potentially leading to a successful university life. This study can be used as a basis for quality improvement and sustainability of mindfulness-based education programs for university students.

## 1. Introduction

University students experience considerable problems such as grade management, academic burden, conflict with friends, difficulties in social relationships, dissatisfaction with school life, financial difficulties, job-related stress, and anxiety about the future [1]. These negative experiences affect the mental and physical health of university students. When faced with these unfavorable experiences, if they do not manage their problems properly, it will be difficult for them to lead a successful university life, and their quality of life will drop. In particular, Korea has the highest suicide rate among OECD countries [2]. Suicide is reported as the biggest cause of death in young adults in their 20s, an age group including many university students [3]. Therefore, educational programs that can promote university students’ emotional stability and psychological well-being are becoming mandatory to an increasing degree in Korea.

Previous literature has shown that maintaining mindfulness is important for students to have a successful university experience [4,5,6]. Mindfulness is defined as “the awareness that emerges through paying attention on purpose, in the present moment, and nonjudgmentally to the unfolding of experience moment by moment” [7] (p. 145). Kabat-Zinn [8] introduced a mindfulness-based stress reduction (MBSR) program to reduce individuals’ psychological symptoms and reported positive effects. Along with the effective results of MBSR in enhancing individuals’ well-being [9], it has been found that mindfulness is connected to low levels of anxiety and depressive symptoms, as well as high levels of self-esteem and life satisfaction [10]. Accordingly, the use of mindfulness-based practices is becoming more popular in many fields such as medicine [9,11], psychology [12,13,14], and education [15,16,17]. These studies have found mindfulness-based practices effective for people to reduce stress. In Korea, research on the effects of mindfulness practices on psychological and physical aspects has been rapidly increasing among adults, including university students [18,19,20]. However, studies of mindfulness practice’s influence on brain waves (central nervous system; CNS) and the autonomic nervous system (ANS) among Korean university students are still lacking. Considering that university students could feel emotionally unstable due to a variety of sources of stress but still should possess good judgment and excellent grades for their future, efficient brain functions are critical. As mindfulness interventions affect the emotions along with memory, applying a mindfulness-based program to university students will not only promote their mental health but also improve cognitive functions.

Brain waves are small wave phenomena that include a wide low-frequency range of 0.1 to 80 Hz occurring in the brain. They are generally measured through the scalp electroencephalogram (EEG) attached to scalp electrodes. The EEG is an effective method to evaluate the CNS functions by measuring physiological changes in the brain in an objective, non-invasive, and continuous manner [21]. Among brain waves, alpha, beta, and theta waves have been recognized as important biological signatures to evaluate a successful mindfulness state [22]. The alpha wave (8–13 Hz) is a fundamental wave that reflects the neuro-physiologically stable state of the brain; it is associated with a relaxed state, stress reduction, concentration, and memory improvement [23]. On the other hand, the beta wave (13–30 Hz) appears predominantly in the active state. When the beta wave is generated, central nerves consume a lot of energy; if this state persists, the brain feels limited in information processing or reactions and lacks concentration [24]. It has also been reported that the beta wave notably emerges when people experience anxiety and stress due to external stimuli [25,26]. Additionally, the theta wave (4–8 Hz) plays a key role in learning and memory, creativity, intuitive understanding [27], promoting stress relief, deep relaxation, and meditation [28]. In fact, an elevated theta wave is related to modifications in CNS arousal often observed during meditation [29,30]. Systematic reviews have identified that mindfulness-based practice is most commonly related to increased alpha and theta brain waves [22,29]. In addition, there is a plethora of studies on mindfulness and EEG noting a decreased beta wave during meditative mindfulness practices [31,32,33,34].

The ANS consists of a sympathetic nervous system (SNS) and a parasympathetic nervous system (PNS). When the SNS is activated, the heart rate and the amount of blood flowing to the muscles increase, while the amount of blood flowing to the skin decreases; this generates a state of tension in the human body [34]. In comparison, when the PNS is activated, heart rate is stabilized, breathing is smooth, and blood pressure decreases, which leads to a relaxed state [35]. Irregular beat-to-beat fluctuations are usually caused by the interaction of the SNS and PNS, which control the cardiovascular function [36]. The change in heart rate is called heart rate variability (HRV). HRV reflects both the SNS and PNS significantly, and it is an objective and non-invasive method to evaluate ANS functions on heart activities [37]. Thus, HRV is one of the valuable parameters for psychological well-being, most commonly described by low frequency (LF; 0.04–0.15 Hz) and high frequency (HF; 0.15–0.40 Hz); specifically, an increase in the HF is related to PNS activation, and the LF reflects SNS activities [37]. The literature has discussed that an unstable emotional state, or stress, raises the beta waves and heart rate but lowers HRV; in contrast, mindful practice leading to relaxation enhances alpha and theta waves but reduces heart rate, which increases HRV [38]. This may help people maintain a calm and comfortable mental state and consequently improve their level of mindfulness. The psychological state and the physiological state are closely connected with each other; that is, when the psychological state changes, the physiological state can also change, and vice versa [38].

This study aimed to identify the effects of a mindfulness-based education program on brain waves, ANS, and the level of mindfulness among the students who participated in the program. Based on the previous literature, we proposed the following hypotheses:

**Hypothesis** **1** **(H1).** There will be a change in brain waves among the participants of the mindfulness-based education program; the alpha and theta waves will increase, but the beta wave will decrease after the intervention.

**Hypothesis** **2** **(H2).** There will be a change in ANS functions among the participants of the mindfulness-based education program; SNS activities will decrease, but PNS activities will increase after the intervention.

**Hypothesis** **3** **(H3).** The level of mindfulness of the participants will change after the intervention.

There are not many universities implementing mindfulness-based education programs in Korea. Only a few universities have introduced them and are operating them on a pilot basis. The K University, located in G metropolitan city in Korea has designated mindfulness-based subjects as mandatory liberal arts courses since 2015; for the balanced development of major education and extra-curricular education, as well as continuous mindfulness education, a mindfulness-based education program funded by the University Innovation Support Project of the Korean government was developed in 2019. This mindfulness-based education program was based on the mindfulness-related liberal arts courses that had been implemented since 2015 at K University. The program was first applied in 2019, pilot applied as online videos in 2020 due to the COVID-19 pandemic, and conducted in the form of real-time online classes in 2021 as intervention of the present study. We attempted to comprehensively identify the effects of the mindfulness-based education program on the mindfulness, brain waves, and ANS among university students. Furthermore, given that most studies on mindfulness with brain waves and ANS have been separately conducted [29,37,39], we endeavored to include both brain waves and ANS as well as mindfulness-scale quantitative data to expansively investigate the effects of the mindfulness-based education program. This study can be used as a basis for quality improvement and sustainability of mindfulness-based education programs for university students.

## 2. Materials and Methods

### 2.1. Research Design

**Figure 1:**
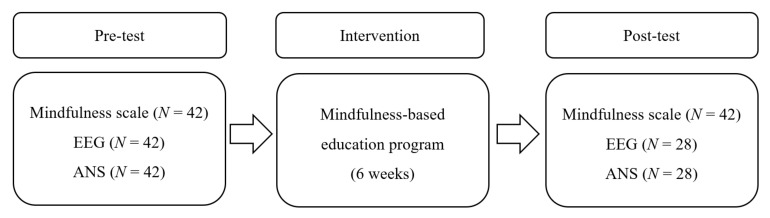
Research design 2.3. Data Collection.

This study is a quantitative and experimental research with a single-group pre–post design (Figure 1). This study was implemented to verify the effects of the mindfulness-based education program at K University on students’ brain waves, ANS, and level of mindfulness.

### 2.2. Participants

The number of participants was calculated using the G*power 3.1.9 software program (University of Düsseldorf, Düsseldorf, Germany) for the paired *t*-test. We applied a medium effect size of 0.50, statistical power of 80.0%, and a significance level of 0.05, revealing a minimum of 34 participants. Considering a 20% attrition rate, a total of 43 students were decided as a sample size. We recruited participants among those who were taking the mindfulness-based education program at K University located in G city in Korea. Participants consisted of second-, third-, and fourth-year undergraduate students; they understood the purpose of the study and consented to participate. Participants received a reward through a mileage program at the university. In order to protect the vulnerable group, we excluded students who belonged to the authors’ department.

The preliminary test was completed on 42 participants. For the post-test, 14 participants who were unable to visit the university due to the COVID-19 pandemic only completed a questionnaire online. In the questionnaire measuring the degree of mindfulness, 42 participants were analyzed. In the EEG and ANS measurement, 14 participants were dropped from the measurement, so data from a total of 28 participants were analyzed (Figure 1).

To investigate the effects of the mindfulness-based education program applied as an intervention, the participants’ mindfulness, EEG, and ANS functions were measured before and after the intervention. Data were collected between 12 April and 4 June 2021, including the six-week intervention. This study was approved by the institutional review board of K University (1041465-202103-HR-001-09). The two present researchers explained the purpose of the study to the participants, and the volunteering students signed a written consent form to participate in the study. Students were assured that their data would be kept confidential and used only for research purposes. They were also allowed to withdraw from the study any time they wished.

### 2.3. Instruments

#### 2.3.1. Mindfulness

The Me, Another, Us, and Mate (MAUM) Scale 21 [40] was used to measure the level of mindfulness. The MAUM Scale 21 was developed in Korean to evaluate the educational effects of mindfulness-based liberal arts classes at K University in 2020. Based on previous theories and studies related to mindfulness abroad and in Korea [41,42,43,44,45,46], the MAUM Scale 21 was developed by a group of experts consisting of eight professors with rich experiences in mindfulness-based education and research at K University. We used this scale as it demonstrated satisfactory internal reliability and validity, particularly with the students who took mindfulness-based liberal arts classes at K University [40]. This scale includes four sub-categories with 21 items: eight items regarding self-esteem (e.g., I am a person with many strengths), six items on positive thinking (e.g., I expect a lot more good things to come in the future than now), three items on resilience (e.g., I believe that there is always a solution even when bad things happen), and four items on self-understanding (e.g., I am well aware of my emotions felt at the present moment). It uses a 5-point Likert scale ranging from 1 for “strongly disagree” to 5 for “strongly agree.” The internal reliability of Cronbach’s α was 0.950 at the time of development and 0.948 in this study. Cronbach’s alphas in sub-categories were 0.923, 0.835, 0.693, and 0.732 for self-esteem, positive thinking, resilience, and self-understanding, respectively.

#### 2.3.2. Electroencephalogram (EEG)

EEG measurements were made with a wireless dry EEG device DSI-24 (Wearable Sensing, San Diego, CA, USA). Before measuring, participants rested sufficiently in a quiet place. Then, EEG measurements were taken with participants in a stable state and with their eyes closed for three minutes in a separate place. The measurement site was maintained at an appropriate indoor temperature and humidity, and body movements were minimized.

For EEG recording, the sensors were accurately positioned based on the start and end times of the brain waves after putting the DSI-24 device on the head; each sensor was placed in close contact with the scalp using a tool that can part the hair. Through this process, the measurement started when the impedance values of all sensors fell below 1 M and the EEG data came out clean for more than 10 s. EEG measurements were taken in a total of 19 areas on the scalp in a monopolar induction method. To acquire signals, a real-time data collection program (DSI-streamer version 2.3 (Wearable Sensing, San Diego, CA, USA) using Dry Cap was utilized, according to the 10/20 international electrode arrangement method. The participants’ EEG signals, received from 19 channels, were stored in a computer as a 16-bit analog-digital conversion at a 300 Hz sampling frequency and a 0.003–150 Hz frequency passband.

#### 2.3.3. Autonomic Nervous System (ANS)

We assessed the participants’ ANS by measuring HRV with the DSI-24 (Wearable Sensing, San Diego, CA, USA). This device tests the autonomic nerve function using an electrocardiogram test, which is an electrical signal generated by the heart, simultaneously measuring the EEG. The participants were instructed not to overwork or drink alcohol the day before the measurement. They were also told not to consume caffeinated beverages, cigarettes, or medications three hours before the measurement and not to do a workout just before the measurement. Before the measurement, the participants were allowed to have sufficient rest in a quiet space. Then, electrodes were attached to their earlobes and the inside their wrists. After waiting for a few minutes to adapt themselves to the measurement environment, they were measured for about five minutes while sitting comfortably in a chair.

### 2.4. Interventions

The mindfulness-based education program applied as an intervention in this study was developed by K University for the purpose of character education in 2019. Based on the mindfulness-related liberal arts courses that had been conducted since 2015 at K University, the professors who had taught those courses for four years were involved in the program development. The program intervention period was set at six weeks based on a previous meta-analysis on the effects of mindfulness meditation programs with university students [47]. Furthermore, by consulting the results of meta-analysis, we confirmed that the length of mindfulness-based training periods varied between four weeks and 24 weeks, and most training periods conducted a 45 min session once a week [48]. Thus, the number of six sessions in this study, with each session being two hours long, was assumed to have meaningful results. The intervention was conducted by three expert professors in mindfulness-based education in a Zoom online class, considering the COVID-19 situation in Korea. Before applying the intervention, three experts tried to maintain consistency conducting the class through sufficient prior discussions. They also had regular meetings to discuss the class operation with identical contents during the intervention period.

The goals of the K University mindfulness-based education program are (1) to cultivate ability as a mindfulness process facilitator and meditation coach through an understanding of mindfulness and meditation practice, (2) to develop the capacity of coordinators, class atmosphere creators, and observers in groups through group activities that lead to self-understanding, communication, empathy, and gratitude, and (3) to improve competence as a facilitator of listening and communication through interaction among members. The teaching methodology of the program is called “MAUM-based active learning”; students learn the theory first and experience not only practical education focusing on meditation, but also “expansion” to find educational content learned in daily life, and “practice” to share examples of actual mindfulness practices [49]. The detailed contents of the six-session program are presented in Table 1.

### 2.5. Data Analyses

All analyses were performed using SPSS 23.0 (IBM Corporation, Armonk, NY, USA). The study variables were analyzed with paired-sample *t*-tests. The mapping for the results was analyzed using TeleScan (Ver 3.2, Laxtha, Daejeon, Korea). The specific methods for analyses are as follows:

- The general characteristics of the participants were analyzed by frequency, percentage, mean, and standard deviation.
- Differences in mindfulness, brain waves, and the ANS before and after the intervention were analyzed by paired *t*-test.
- The results of each EEG channel, which showed significant differences before and after the intervention, were visualized through mapping.
- EEG signals (RawData) were analyzed after converting the original file into a time series analysis program. In particular, the relative power of brain waves was used for analysis based on the previous findings that relative power measures demonstrated better estimates for brain waves [29,50].

## 3. Results

### 3.1. General Characteristics of the Participants

Table 2 displays the general characteristics of the participants. The average age was 20 years old. The participants consisted of 21 second-year students (50.0%), 15 third-year students (35.7%), and 6 fourth-year students (14.3%). Regarding their majors, 17 students (40.5%) were majoring in humanities and social sciences, 11 students (26.2%) were majoring in the health sciences, 9 (21.4%) were majoring in natural sciences, 3 (7.1%) were in the arts and physical education department, and 2 (4.8%) belonged to the teacher education department.

### 3.2. Mindfulness

To examine differences in mindfulness the paired *t*-test was conducted, and the results are shown in Table 3. As hypothesized, there was a significant difference in the level of students’ mindfulness before and after participating in the mindfulness-based education program (*t* = −2.95, *p* = 0.005), with a mean change from 3.72 ± 0.73 to 3.89 ± 0.81. In terms of the mindfulness sub-categories, there was a significant difference in self-esteem before intervention 3.32 ± 0.90 and after intervention 3.60 ± 1.04 (*t* = −3.31, *p* = 0.002) and in self-understanding before intervention 3.56 ± 0.77 and after intervention 3.89 ± 0.76 (*t* = −3.03, *p* = 0.004). There was no significant difference in positive thinking or resilience levels.

### 3.3. Electroencephalogram (EEG)

#### 3.3.1. Relative Alpha, Relative Beta, and Relative Theta Waves

As university classes were switched to online classes due to the COVID-19 situation in Korea, 14 out of the total 42 students who were unable to perform the in-person EEG test and the ANS post-measurement were dropped from the measurement. Thus, data from the final 28 students were compared and analyzed. Table 4 displays the EEG results showing a significant difference before and after applying the intervention program.

Alpha waves and theta waves significantly increased in channel 1 (*t* = −2.11, *p* = 0.022), 2 (*t* = −1.91, *p* = 0.034), 7 (*t* = −3.39, *p* = 0.001), and 14 (*t* = −2.20, *p* = 0.019) for alpha waves; and channel 9 (*t* = −1.99, *p* = 0.028), 10 (*t* = −2.26, *p* = 0.016), 12 (*t* = −2.24, *p* = 0.017), and 19 (*t* = −1.76, *p* = 0.045) for theta waves after the intervention. In comparison, beta waves decreased in channel 1 (*t* = 1.98, *p* = 0.029), 2 (*t* = 2.60, *p* = 0.008), 10 (*t* = 1.89, *p* = 0.035), 12 (*t* = 1.79, *p* = 0.043), 13 (*t* = 1.82, *p* = 0.040), 14 (*t* = 2.55, *p* = 0.009), and 17 (*t* = 2.20, *p* = 0.018); significantly decreased beta waves were observed overall in the entire brain. The results generally support our hypothesis of increased alpha and theta waves, but the beta wave decreased after the intervention.

#### 3.3.2. Mapping

**Figure 2:**
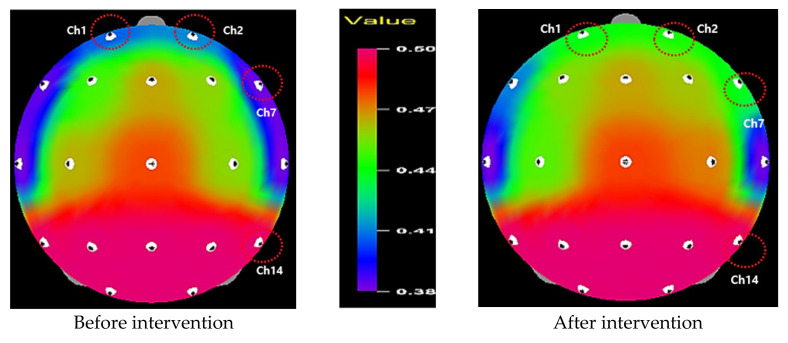
Relative alpha wave.

**Figure 3:**
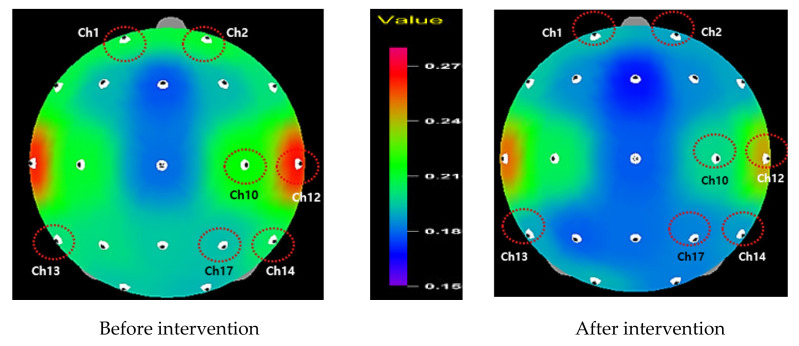
Relative beta wave.

**Figure 4:**
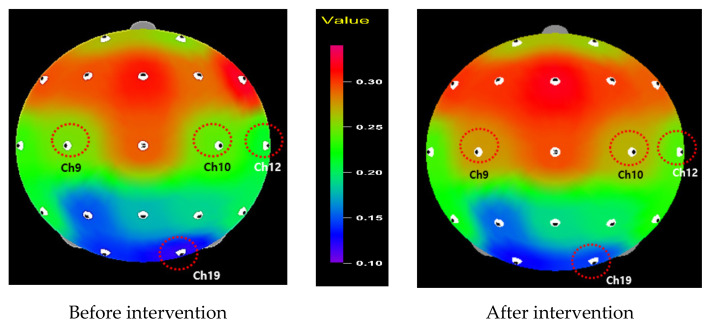
Relative theta wave.

Figure 2, Figure 3 and Figure 4 display the mappings of EEG results showing significant differences before and after program application. Alpha waves significantly increased in channels 1, 2, 7, and 14, which were located in the pre-frontal lobe, frontal lobe, and temporal lobe areas (Figure 2). In contrast, beta waves significantly decreased in channels 1, 2, 10, 12, 13, 14, and 17, demonstrating an overall decrease in the entire brain (Figure 3). Finally, theta waves significantly increased in channels 9, 10, 12, and 19, which were located in the central lobe, temporal lobe, and occipital lobe areas (Figure 4).

### 3.4. Autonomic Nervous System (ANS)

There was no significant change in ANS functions among the participants. Specifically, the result did not show any differences in HF or LF before and after the intervention, presenting no significant HRV change (Table 5).

## 4. Discussion

This study investigated the effects of a mindfulness-based education program on brain waves, ANS, and mindfulness among university students who participated in a six-week intervention.

First, we found that the alpha and theta waves increased, but the beta waves decreased after the six-week intervention of the mindfulness-based education program, supporting Hypothesis 1. These results are consistent with previous findings in several studies [22,29,31,32,39]. For the alpha waves, channels 1 and 2 (pre-frontal lobe), channel 7 (frontal lobe), and channel 14 (temporal lobe) were enhanced. In particular, the increased alpha-wave activity on the left side of the pre-frontal lobe (channel 1) is meaningful, in that the increased activity in the left hemisphere is related to lower anxiety, higher positive emotions, and the perception of well-being [51,52]. Our result indicates that the mindfulness-based program could promote students’ positive and relaxed emotions. This corroborates the Functional-MRI finding that the left-side pre-frontal lobe was activated when people were optimistic and passionate, or experiencing positive emotions [53]. The increased alpha power after the mindfulness-based program also supports the theoretical notion that higher alpha power indicates a relaxed mental state by improving attentional awareness [54,55]. This reflects that mindfulness-based meditation is a systematic practice of attention and self-control capacities [39].

For the theta waves, channels 9 and 10 (central lobe), channel 12 (temporal lobe), and channel 19 (occipital lobe) were enhanced. The literature has suggested that increased theta-wave activity is connected to changes in CNS arousal, which is often found in mindfulness-based meditation practitioners [30,56]. Along with the increased alpha activity in the present study, the increased theta activation also indicates that the higher level of theta power could induce changes in attentional distribution and positive emotions, as supported by earlier studies [29,57,58]. Finally, for the beta waves, channels 1, 2, 10, 12, 13, 14, and 17 were reduced overall in the brain area. Beta brain waves are often related to high arousal and cognitive energy consumption; when this status persists, brain activity ultimately becomes slower in information processing or in responses [24]. Moreover, it has been consistently reported that high beta power is associated with anxiety and stress [25,26]. However, beta waves were reduced during the practice of mindful meditation [31,32,33,34], suggesting that mindfulness-based practice leads to a relaxation status.

As countless studies have discovered increased alpha and theta waves, as well as decreased beta waves among participants in mindfulness-based meditation [22,29,30,56], our consistent results suggest that the participants in this study were supported in managing a relaxed status after the six-week mindfulness-based education program, despite many kinds of potential stress sources in their life. Specifically, our result of anterior activation described by the increased alpha activity implies that the mindfulness-based education program can potentially promote positive emotions. Considering the correlation between active pre-frontal lobe and positive emotions [51,52], future research could expand the study on the relationships between brain waves and emotions. Furthermore, our results provide an opportunity to better understand the psychophysiological nature of mindfulness-based meditation.

Second, we hypothesized that there would be lower SNS activity and higher PNS activity after the intervention, expecting an increase in HRV. However, this hypothesis was not supported, presenting non-significant changes in HF and LF before and after intervention. The reason for this may be due to the small sample size in the present study. As Ivaki et al. [36] has suggested, in future research, larger sample sizes would be needed to capture the effect of mindfulness-based interventions in the ANS. Additionally, some studies reported inconsistent results of the HRV factors in meditative states [34,59,60]. Other physiological factors, such as participants’ circadian rhythm, respiration, or body posture, might have also influenced the non-significant result, also discussed in previous studies [37,61]. Furthermore, positive emotions such as love, appreciation, and compassion are reflected by the HRV index [62,63]; therefore, it is worthwhile to conduct further repetitive studies to identify the significant effect of mindfulness-based interventions in the ANS, focusing on positive and negative emotions.

Meanwhile, the literature has discussed that brain waves and heart rate are correlated. For example, alpha and theta waves increased in the frontal lobe during meditation, along with an increase in HF and a decrease in LF in HRV [64]. Moreover, when the cardiac coherence index measured by HRV increased during meditation, the absolute alpha wave measured in the parietal lobe significantly increased, and the relative alpha wave also increased overall in the brain area [34]. These findings support the dynamic correlation between brain waves and HRV activity. However, our study could not identify a correlation between them due to a non-significant HRV change before and after the intervention. Future studies might investigate this correlation more thoroughly to understand the mechanism by which the brain and peripherals interact when combining both the brain waves and peripheral indices.

Lastly, the level of mindfulness increased in the participants after the intervention, supporting Hypothesis 3. Our results are in line with earlier findings that mindfulness-based meditation interventions reinforced participants’ self-reported mindfulness [65,66,67]. The increased mindfulness corroborates that mindfulness is closely related to physical and psychological well-being by reducing mental and physical symptoms of stress [10,68]. Specifically, among the four sub-categories of mindfulness, self-esteem and self-understanding significantly improved. This result is supported by Brown and Ryan’s [10] finding that self-reported mindfulness was positively associated with self-esteem and life satisfaction among university students and general adults. In addition, the increased self-understanding may explain that awareness of thoughts, emotions, and physical sensations, which are main characteristics of mindfulness, may have helped the students to better understand what they were experiencing. This is, in fact, nicely coordinated with the presence of an increased alpha wave, indicating a relaxed mental state by improving attentional awareness [54,55]. Regarding no significant increase in the sub-categories of positive thinking and resilience, one possible reason for this could be the online class format; students might have been less focused on meditation in their online class, compared to an in-person class. Another potential reason might be concerned with the use of the MAUM Scale 21 to evaluate mindfulness. Unlike widely used mindfulness measures such as the Mindful Attention and Awareness Scale (MAAS) [10] or the Cognitive and Affective Mindfulness Scale-Revised (CAMS-R) [69], the MAUM Scale 21 has not been tested in other studies to date. Although the MAUM Scale 21 was developed and validated by the experts who were profoundly involved in mindfulness-based liberal arts classes at K University, it might have not accurately captured mindfulness among the participants. More extensive application of the MAUM Scale 21 is needed in the future to firmly establish the scale to measure mindfulness.

This study has some limitations to consider. First, the sample included only female students, and the size was small. In fact, previous literature claims that gender plays a key role in the benefits of meditation-based therapies [70]. The study is therefore limited in its ability to generalize the results for both male and female students. Future research should explore this type of mindfulness-based program in larger and varied samples that include both genders to see if the findings are replicable. Second, this study did not include a control comparison group, which makes it difficult to claim strong conclusions of positive effects of intervention. Inclusion of a control group in the future can make stronger inferences when implementing the mindfulness-based education program and evaluating the effects. Third, our mindfulness intervention program consisted of six sessions; even though each session lasted for two hours, it could be considered a smaller number of sessions than in previous programs, given the average of eight sessions reported in the meta-analysis [48]. Future research might conduct more sessions to thoroughly evaluate the effects of the mindfulness-based education program. Lastly, we did not conduct follow-up assessments on the effects of the program; thus, it is limited in its ability to guarantee any long-term effects of the program. Just as regular workout is necessary to keep physical health, sustaining mindfulness practices is critical to constantly receive benefits of mindfulness for psychological well-being. Follow-up studies are strongly recommended to assess the impact of training over time.

Despite the limitations, the present study does make contributions to the field of mindfulness research. First, this study is one of the few studies that included both central and peripheral activities to identify the effect of a mindfulness-based program by simultaneously measuring brain waves and ANS. Furthermore, we added quantitative data through the administration of a mindfulness questionnaire to comprehensively investigate the effects of the intervention program on top of the data on CNS and ANS functions. Second, this study is innovative in that we made an effort to establish the mindfulness-based education program in the curriculum and identify the effects that it has had, in a general environment where there exists little interest in mindfulness education in most Korean universities. This study emphasizes the importance of character education within a mindfulness-based education program. Based on the present study, we can constantly strive to improve the program’s quality and sustain an effective mindfulness-based education program for university students, hopefully leading to a curricular change in many universities.

## 5. Conclusions

We identified the positive effects of the mindfulness-based education program for university students. Specifically, after participating in the six-week mindfulness-based intervention, students demonstrated significant increases in both alpha and theta brain waves but decreases in the beta wave. In addition, the level of mindfulness improved after participating in the program. These findings support the relaxation effect of the mindfulness-based education program in university students; this program may enhance students’ positive emotional experiences combined with developed attentional awareness. In fact, mindfulness is a process that makes people calmer and more relaxed, yet more wakeful, simultaneously [28]. We believe that this program may help students not only relax, but also generate a mindfulness state in stressful situations. Ultimately, university students can benefit from this program by creating positive learning experiences and thereby promote higher satisfaction with their university life.

## Figures and Tables

**Table 1:** The Contents of the Six-Week Program.

Week	Topic	Contents
1	Orientation, Basic understanding of mind and mindfulness	Self-introduction, understanding of mindfulness guidance, and program introduction Basic understanding of mind and mindfulness
2	Mindfulness and meditation	Mindfulness methods and effects Mindfulness meditation and sitting meditation
3	Meditation practice	Loving-kindness meditation, eating meditation, and walking meditation Body scan and music meditation
4	Self-understanding and acceptance	Looking into the state of mind as it is Mind discovery, self-understanding, and self-expression
5	Communication, empathy, and gratitude	Understanding and respect for differences in others and myself Listening and empathic conversation Finding gratitude in daily life
6	Practice of meditation guidance	Practice of meditation guidance: Sharing my meditation practice Presentation of practice cases of mindfulness Giving thoughts, reflection, and sharing

**Table 2:** General Characteristics of the Participants (*n* = 42).

Categories		*n* (M)	% (SD)
Age (years)		20.07	1.02
Year in School	Second	21	50.0
	Third	15	35.7
	Fourth	6	14.3
Major	Humanities and Social Sciences	17	40.5
	Health Sciences	11	26.2
	Natural Sciences	9	21.4
	Arts and Physical Education	3	7.1
	Teacher Education	2	4.8
Religion	Protestant	7	16.7
	Catholic	3	7.1
	Buddhist	1	2.4
	Unidentified	1	2.4
	No religion	23	54.8
	No response	7	16.7

Note: Data presented as number (*n*), percentage (%); M = mean, SD = standard deviation.

**Table 3:** Differences in Mindfulness (*n* = 42).

Variables	Pre	Post	*t*	*p*
	M ± SD			
Mindfulness	3.72 ± 0.73	3.89 ± 0.81	−2.95	0.005 **
Self-esteem	3.32 ± 0.90	3.60 ± 1.04	−3.31	0.002 **
Positive thinking	4.04 ± 0.76	4.09 ± 0.78	−0.71	0.480
Resilience	4.24 ± 0.63	4.29 ± 0.76	−0.62	0.538
Self-understanding	3.56 ± 0.77	3.89 ± 0.76	−3.03	0.004 **

Note: ** *p* < 0.01.

**Table 4:** Differences in Brain Waves (*n* = 28).

Variables	Pre	Post	*t*	*p*
	M ± SD			
RA				
Ch 1 (pre-frontal L)	0.41 ± 0.15	0.44 ± 0.16	−2.11	0.022
Ch 2 (pre-frontal L)	0.41 ± 0.15	0.44 ± 0.17	−1.91	0.034
Ch 7 (frontal L)	0.38 ± 0.15	0.44 ± 0.14	−3.39	0.001
Ch 14 (temporal L)	0.58 ± 0.14	0.61 ± 0.14	−2.20	0.019
RB				
Ch 1 (pre-frontal L)	0.21 ± 0.08	0.19 ± 0.08	1.98	0.029
Ch 2 (pre-frontal L)	0.21 ± 0.09	0.19 ± 0.08	2.60	0.008
Ch 10 (central L)	0.22 ± 0.08	0.20 ± 0.08	1.89	0.035
Ch 12 (temporal L)	0.26 ± 0.09	0.24 ± 0.08	1.79	0.043
Ch 13 (temporal L)	0.19 ± 0.09	0.18 ± 0.09	1.82	0.040
Ch 14 (temporal L)	0.20 ± 0.09	0.18 ± 0.09	2.55	0.009
Ch 17 (parietal L)	0.20 ± 0.09	0.19 ± 0.09	2.20	0.018
RT				
Ch 9 (central L)	0.25 ± 0.06	0.28 ± 0.09	−1.99	0.028
Ch 10 (central L)	0.24 ± 0.06	0.27 ± 0.07	−2.26	0.016
Ch 12 (temporal L)	0.21 ± 0.09	0.24 ± 0.08	−2.24	0.017
Ch 19 (occipital L)	0.12 ± 0.05	0.14 ± 0.09	−1.76	0.045

Note: RA = relative alpha, RB = relative beta, RT = relative theta, Ch = channel, L = lobe. Only significant changes are presented.

**Table 5:** Differences in Heart Rate Variability (HRV) (*n* = 28).

Variables	Pre	Post	*t*	*p*
	M ± SD			
LF	0.44 ± 0.18	0.45 ± 0.19	−0.23	0.409
HF	0.56 ± 0.18	0.55 ± 0.19	0.23	0.409
HR	79.97 ± 9.90	77.53 ± 11.44	1.59	0.062

Note: LF = low frequency, HF = high frequency, HR = heart rate/min.

## Data Availability

The data presented in this study are available upon request from the corresponding author.
